# Draft genome sequence of an *Enterococcus camelliae* strain MBBL_MP6 isolated from a crossbred Holstein Friesian cow diagnosed with clinical mastitis

**DOI:** 10.1128/mra.00497-26

**Published:** 2026-09-29

**Authors:** Kh. Yeashir Arafat, Md. Anamul Kabir Hemal, Sourav Karmakar, Ziban Chandra Das, Tofazzal Islam, M. Nazmul Hoque

**Affiliations:** 1 Molecular Biology and Bioinformatics Laboratory, Department of Gynecology, Obstetrics and Reproductive Health, Gazipur Agricultural University 198780, Gazipur, Bangladesh; 2 Institute of Biotechnology and Genetic Engineering, Gazipur Agricultural University 198780, Gazipur, Bangladesh; University of Maryland School of Medicine, Baltimore, Maryland, USA

**Keywords:** *Enterococcus camelliae*, mastitis milk, dairy cow, draft genome

## Abstract

We report the draft genome sequence of *Enterococcus camelliae* strain MBBL_MP6, isolated from bovine clinical mastitis milk. The genome assembly comprises 62 contigs, totaling 2,350,649 bp with 38.5% GC content, and 190× coverage. Genome analysis revealed no virulence factor gene and only one antimicrobial resistance gene.

## ANNOUNCEMENT

*Enterococci* are gram-positive lactic acid bacteria commonly inhabiting the gastrointestinal tracts of humans and animals, as well as food and environmental sources (1, 2). Depending on the context, they may act as commensals, opportunistic pathogens, or beneficial agents in fermentation (3–5). Among them, *Enterococcus camelliae* has been primarily associated with plant and food-related environments, particularly tea plants and fermented tea products (6). Based on the available literature, this is the first report describing the isolation of *E. camelliae* from bovine mastitis milk. Therefore, comprehensive genomic characterization of newly identified isolates is essential to elucidate their metabolic potential, antimicrobial resistance determinants, virulence-associated features, and prospective applications in agriculture and food processing. In this study, we report the draft genome sequence of *E. camelliae* strain MBBL_MP6, isolated from milk of a Holstein Friesian crossbred dairy cow with clinical mastitis at a commercial farm in Gazipur, Bangladesh (24.09582° N, 90.41252° E), in July 2025. Clinical mastitis was confirmed using the California Mastitis Test (7), and a milk sample was collected aseptically and transported to the laboratory under chilled conditions (4°C) (4, 8). For enterococcal isolation, a 100 µL aliquot of milk was inoculated into 5 mL of nutrient broth (HiMedia, India) and incubated aerobically overnight at 37°C. Following incubation, a 10^−^³ serial dilution was prepared, and a 10 µL aliquot was streaked onto Columbia agar (HiMedia, India) supplemented with 5% sheep blood (9). Species-level identification was carried out using the VITEK 2 system v9.01 (10). Genomic DNA was extracted from an overnight nutrient broth culture of strain MBBL_MP6 using the QIAamp DNA Mini Kit (QIAGEN, Germany). Sequencing libraries were prepared from 1 ng of DNA with the Nextera DNA Flex Kit (Illumina, USA) and sequenced on the Illumina MiSeq platform in a 2  ×  250 bp paired-end configuration (11), yielding 2,028,206 raw reads. Read quality was assessed with FastQC v0.11.3 (12), and adapter trimming along with low-quality read filtering was performed using Trimmomatic v0.39.2 (13). Genome completeness was evaluated with CheckM v1.2.4 (14), which estimated 95.73% completeness with 2.07% contamination. Antimicrobial resistance genes were screened using CARD v4.0.1 (15), which identified a single ARG, while VFDB v6.0 (16) detected no virulence factor genes. PathogenFinder2 v0.5.0 (17) assigned a pathogenicity score of 0.601. CRISPRCasFinder v2.0.3 (18) revealed one CRISPR array, and functional annotation via the RAST v2.0 server (19) predicted 230 metabolic subsystems. All analyses were conducted using default parameters unless otherwise specified.

The genomic features of *E. camelliae* MBBL_MP6 are summarized in Table 1. Despite the presence of only one antimicrobial resistance gene, the pathogenicity score of 0.601 suggests that this isolate may function as an opportunistic pathogen capable of causing mastitis in dairy animals.

**TABLE 1:** Genomic features of *Enterococcus camelliae* MBBL_MP6 isolated from bovine mastitic milk of dairy cows

Genomic features	*E. camelliae* MBBL_MP6
GenBank accession	JBWNLF000000000
BioSample accession	SAMN53127716
Assembled genome size (bp)	2,350,649
GC %	38.5
Coverage (×)	190×
*N*_50_ value (bp)	71,009
Number of contigs	62
Genome completeness	95.73%
Genome contamination	2.07%
Genes (total)	2,389
Coding sequences (CDSs; total)	2,319
Genes (coding)	2,261
CDSs (with protein)	2,261
Genes (RNA)	70
Ribosomal RNAs	4, 5, 1 (5S, 16S, 23S)
Complete ribosomal RNAs	3, 1, 1 (5S, 16S, 23S)
Partial ribosomal RNAs	1, 4 (5S, 16S)
Transfer RNAs	56
Non-coding RNAs	4
Pseudogenes (total)	58
CDSs (without protein)	58
Pseudogenes (ambiguous residues)	1 of 58
Pseudogenes (frameshifted)	20 of 58
Pseudogenes (incomplete)	34 of 58
Pseudogenes (internal stop)	21 of 58
Pseudogenes (multiple problems)	14 of 58
ARG	1
VFG	0
CRISPR arrays	1
Number of subsystems	230
PathogenFinder score	0.601
Input proteome coverage (%)	0.22
Matched pathogenic families	3
Matched not pathogenic families	2

## Data Availability

The whole-genome project of *Enterococcus camelliae* MBBL_MP6 has been deposited in GenBank under accession number JBWNLF000000000 and version JBWNLF000000000.1 and is associated with BioProject accession number PRJNA1358723. The raw sequencing reads have been submitted to the National Center for Biotechnology Information Sequence Read Archive under accession number SRR39451540.
